# Synthesized Geopolymers Adsorb Bacterial Proteins, Toxins, and Cells

**DOI:** 10.3389/fbioe.2020.00527

**Published:** 2020-06-03

**Authors:** John Popovich, Shaojiang Chen, Natalie Iannuzo, Collin Ganser, Dong-Kyun Seo, Shelley E. Haydel

**Affiliations:** ^1^Center for Immunotherapy, Vaccines and Virotherapy, The Biodesign Institute, Arizona State University, Tempe, AZ, United States; ^2^School of Molecular Sciences, Arizona State University, Tempe, AZ, United States; ^3^School of Life Sciences, Arizona State University, Tempe, AZ, United States; ^4^School of Earth and Space Exploration, Arizona State University, Tempe, AZ, United States; ^5^Center for Molecular Design and Biomimetics, The Biodesign Institute, Arizona State University, Tempe, AZ, United States; ^6^Center for Bioelectronics and Biosensors, The Biodesign Institute, Arizona State University, Tempe, AZ, United States

**Keywords:** aluminosilicate, geopolymer, nanoporous, adsorption, adsorbent, toxin removal, bacteria

## Abstract

Pore-forming and hemolytic toxins are bacterial cytotoxic proteins required for virulence in many pathogens, including staphylococci and streptococci, and are notably associated with clinical manifestations of disease. Inspired by adsorption properties of naturally occurring aluminosilicates, we engineered inexpensive, laboratory-synthesized, aluminosilicate geopolymers with controllable pore and surface characteristics to remove pathogenic or cytotoxic material from the surrounding environment. In this study, macroporous and mesoporous geopolymers were produced with and without stearic acid surface modifications. Geopolymer binding efficacies were assessed by measuring adsorption of methicillin-resistant *Staphylococcus aureus* (MRSA) culture filtrate proteins, α-hemolysin and streptolysin-O toxins, MRSA whole cells, and antibiotics. Macroporous and mesoporous geopolymers were strong non-selective adsorbents for bacterial protein, protein toxins, and bacteria. Although some geopolymers adsorbed antibiotics, these synthesized geopolymers could potentially be used in non-selective adsorptive applications and optimized for adsorption of specific biomolecules.

## Graphical Abstract

Visual representation of geopolymers (represented by a sponge for their porous properties). MRSA cells, hemolysin (PDF: 7AHL), and other toxins and may bind within or between pores of the geopolymer matrix. Geopolymer surfaces are non-coated or functionalized with stearic acid molecules, as shown.

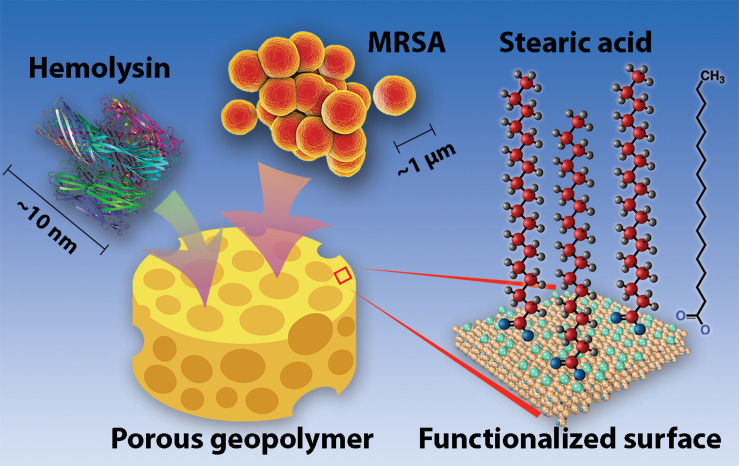

## Introduction

Antibiotic resistant bacterial infections, now originating in both healthcare and community settings (Kallen et al., 2010; Mediavilla et al., 2012; Gross et al., 2014; Blair et al., 2015; Cornejo-Juarez et al., 2015; Martens and Demain, 2017), pose serious consequences for public health and burden the United States economy with up to $20 billion in healthcare costs each year (Golkar et al., 2014). The antibiotic resistance global health crisis is further exacerbated by the worldwide overuse of antibiotics and the concomitant decline in the discovery of new and effective antibiotics (Bartlett et al., 2013; Ventola, 2015a,b). Clinical use of new antibiotics will likely lead to eventual resistance. Hence, exploring and developing alternative therapies that circumvent resistance pressures and can be used in clinical applications are critical.

While bacteria infect nearly all host tissues, skin and soft tissue infections (SSTIs), wound infections, invasive bloodstream infections, and urinary tract infections are particularly frequent (Fischbach and Walsh, 2009; Woodford and Livermore, 2009). Bacteria that cause these infections possess many secreted virulence factors necessary for survival, evasion of the host immune system, and pathogenesis (Gonzalez et al., 2008). A common attribute among prominent bacterial pathogens (e.g., *Staphylococcus aureus*, *Streptococcus pneumoniae*, and *Escherichia coli*) that have developed multidrug resistance is the production of secreted pore-forming cytotoxins (Alouf, 2003; Gonzalez et al., 2008; Los et al., 2013). While pore-forming toxins damage host cell membranes, these toxins also disrupt host immune responses and epithelial/endothelial barriers, highlighting the importance of these proteins in manifesting pathogenic potential (Los et al., 2013). Of the multitude of virulence factors employed by MRSA, cytolytic toxins represent the largest category (Alouf, 2003; Gonzalez et al., 2008), so developing multifunctional biomaterials which sequester bacterial toxins and cells could serve as an anti-virulence therapeutic for recalcitrant and/or resistant infections.

Capitalizing on its mechanical stability and ability to adsorb biomolecules, aluminosilicate porous materials have been used as antimicrobials (in ion-exchanged forms) and adsorbents to treat infections and to reduce toxins in poultry and livestock (Huwig et al., 2001; Davis, 2002; Otto and Haydel, 2013; Yu et al., 2013; Chen et al., 2014; Samiey et al., 2014; Liu et al., 2017). Toxins secreted by microorganisms can be adsorbed by aluminosilicates (Venkateswerlu and Stotzky, 1992; Tapp and Stotzky, 1995; Grant and Phillips, 1998; Morris et al., 2000), potentially reducing the severity of infection (Clatworthy et al., 2007). Aluminosilicate adsorption is mediated by electrostatic and/or van der Waals forces and influenced by chemical structure, surface characteristics, and pore size/architecture of the material (Corma, 2003; Yu et al., 2013). Aluminosilicate zeolites and geopolymers are produced inexpensively and can be synthetically manipulated to enable control of physicochemical characteristics and purification processes for specific uses (Dinesh et al., 2014; Chen et al., 2017; Moshoeshoe et al., 2017). Importantly, the geopolymers and the associated production processes are benign to the environment, in contrast to common adsorbents, such as activated carbon, which have activation processes with introduce hazardous chemical wastes (Wu et al., 2018). Moreover, the pore size of porous geopolymers can be tuned from nanoporous (<100 nm) to the macroporous range. The geopolymers can be customized with large pore sizes to accommodate micron-sized bacterial cells, in contrast to conventional adsorbents that are limited to the nanoporous range.

Geopolymers (GPs), known as the amorphous counterpart to zeolites, are an emerging class of aluminosilicate materials that have significant commercial and technological potential due to its ease of production and high mechanical and chemical stabilities (Kriven et al., 2004; Davidovits, 2008). GPs have a xerogel-like network structure made up of 20–40 nm-sized nanoparticles (Kriven et al., 2004; Dinesh et al., 2014) that have chemical compositions, local chemical structures, and surface characteristics similar to those of zeolites (Kriven et al., 2004; Nel et al., 2009). In addition, GPs can be specifically tailored to control porosity in meso/macroscale, physicochemical properties, and functionality (Dinesh et al., 2014; Sharma et al., 2015). Porous GPs with exposed nanostructures have been developed for emerging applications such as drug delivery (Jamstorp et al., 2011) catalysis (Sharma et al., 2015), and antibacterial activity (O’Connor et al., 2010). In this study, macroporous GP (macroGP) and mesoporous GP (mesoGP) were synthesized with or without stearic acid (SA) modification (SA-macroGP and SA-mesoGP, respectively) and were examined for the ability to adsorb culture filtrate proteins, pore-forming toxins, bacterial cells, and antibiotics.

## Materials and Methods

### Synthesis of Macroporous Geopolymer (macroGP)

Fumed silica (4.15 g) (Cabot, CA-BO-SIL^®^ EH-5; aggregate particle size: 0.2–0.3 μm; primary size: 5–25 nm) was added to 12.0 mL of KOH (11.6 M) solution and mechanically mixed (IKA^®^ RW 60 digital mixer) at 800 rpm for 30 min to dissolve the silica. Metakaolin [7.64 g (Metamax, BASF; average particle size: 1.3 μm)] was added to the solution and the mixture was stirred again at the same speed for 40 min to form a homogenous fluidic liquid. The chemical composition (wt%) of the metakaolin was SiO_2_: 53.0%, Al_2_O_3_: 43.8%, Na_2_O: 0.23%, K_2_O: 0.19%, TiO_2_: 1.7%, and Fe_2_O_3_: 0.43%. Paraffin oil (Alfa Aesar) was then added to the resin at a 1:1 oil-to-water volume ratio and stirred under the same mixing condition for 15 min, resulting in a homogeneous, but viscous emulsion. The emulsion was transferred to a polypropylene tube and cured in a laboratory oven at 60°C for 24 h. The cured monolithic product was crushed into small pieces (1–2 mm) and subjected to Soxhlet extraction with hexane as the solvent. After the extraction, the product was washed with a copious amount of deionized (DI) water until a neutral pH was achieved (pH ∼7) and dried in a lab oven overnight at 90°C.

### Synthesis of Mesoporous Geopolymer (mesoGP)

An aluminosilicate precursor mixture with a composition of 3.1K_2_O: Al_2_O_3_: 5.5SiO_2_: 66H_2_O was prepared by first adding 6.05 g of KOH pellets and 18.03 g of potassium silicate solution (PQ corporation, H_2_O: 60.8 wt%, K_2_O: 12.65 wt%, SiO2: 26.55 wt%) in 14.55 mL of DI water in a cold bath. Once the KOH pellets were dissolved, the solution was brought to room temperature and added with 5.09 g of the metakaolin. After stirring with the mechanical mixer at 800 rpm for 40 min, a visually homogeneous and free-flowing geopolymer resin was obtained. The resin was then sealed in a 50-mL polypropylene tube and heated at 90°C for 6 h. After heating, the resin was transformed into a homogeneous paste. The solid component in the paste was isolated by repetitive washing with DI water and centrifugation at 6,000 rpm (g-force: ∼2,500 m/s^2^) for 15 min until the supernatant was pH ∼8. After drying at 90°C overnight, the resulting powder product was stored in sealed glass vials at room temperature.

### GP Surface Modifications

Following a previous report (Sakthivel et al., 2013), surface modification of the GP products was carried out by utilizing the esterification reaction between silanol groups on the surfaces of geopolymers and stearic acid. Stearic acid (0.2 g) was dissolved in 25 mL of 0.6 M NaOH solution in a 90°C water bath. After adding 1.0 g of macroGP or mesoGP to the hot solution, the mixture was magnetically stirred for 10 min at 90°C and slowly cooled down to room temperature while agitating. Precipitate products were filtered and washed extensively with DI water to reduce the pH to ∼8.0. The products were dried in a lab oven at 60°C overnight and then placed in another oven at 160°C for 4 h to induce crosslinking of carboxylic acid groups to the surface of the geopolymer. Finally, the dried products were washed with hot toluene multiple times to remove unbound stearic acid and then dried again at 60°C overnight, producing stearic acid-modified macroGP (SA-macroGP) and stearic acid-modified mesoGP (SA-mesoGP).

### GP Characterization

Powder X-ray diffraction (PXRD) patterns of the products were collected on a Bruker D5000 powder X-ray diffractometer (Ni-filtered Cu Kα radiation with a wavelength of 1.5406 Å, operated at 30 kV and 30 mA, VANTEC- position-sensitive detector) at a scan speed of 2.0 degrees/min and a step size of 0.02 degrees 2θ. Scanning electron microscopy (SEM) images of powder samples were collected using an XL30 environmental FEG (FEI) microscope operating at 10 kV acceleration voltage. Transmission electron microscopy (TEM) was carried out using a Titan 80-300 FEG-TEM (FEI Company, Hillsboro, OR, United States) operated at 300 kV with a UltraScan camera. Fourier transform infrared (FT-IR) spectra were recorded using a Bruker IFS66 V/S attenuated total reflection (ATR) FT-IR spectrometer. Carbon–hydrogen–nitrogen (CHN) elemental analyses were performed by employing Perkin-Elmer 2400 Series II CHNS/O Analyzer (Waltham, MA, United States) with a thermal conductivity detector. Brunauer–Emmett–Teller (BET) surface areas were estimated with a Micrometrics ASAP 2020 volumetric adsorption analyzer with nitrogen as the adsorbate at 77 K. Prior to the analysis, samples (about 300 mg) were degassed at 300°C for at least 6 h under a vacuum until a residual pressure of ≤10 μm Hg was reached. Specific surface areas were determined from the BET equation. The *t*-plot method was used to distinguish the contributions from micropores and from the mesopores to the pore volume and surface area. The mesopore volumes were calculated after subtracting the micropore volume from the total pore volume. Pore size distributions were obtained using the Barrett–Joyner–Halenda (BJH) method assuming a cylindrical pore model from the desorption branch of sorption isotherms (Barrett et al., 1951).

### Bacterial Strains and Growth Conditions

MRSA USA300, a Gram-positive spherical bacterium (∼1 μm), was cultured at 37°C in either tryptic soy broth (TSB), tryptic soy agar (TSA), or in a chemically defined medium (CDM) (Supplementary Table S1) based on previously created CDMs (Onoue and Mori, 1997; Bosi et al., 2016). To prepare mid-logarithmic phase cultures, MRSA was cultured from an isolated colony for 17–19 h until growth reached saturation, subsequently diluted 1:40 into fresh TSB, and grown for 2.5 h (OD_600_ = 0.3–0.4) until the culture reached mid-logarithmic phase.

### MRSA Culture Filtrate Proteins (CFPs) Adsorption Assay

MRSA was cultured in TSB from an isolated colony for 17–19 h until growth reached saturation, washed and resuspended twice in CDM, and incubated at 37°C with gentle agitation for ∼10 h. MRSA cultures were passed through a 0.22 μm filter (Millipore), and the supernatant containing the CFPs was concentrated using a 3K cutoff filter (Amicon). Protein concentrations were determined using the BCA Protein Assay Kit (Pierce). CFPs (50 μg/mL) were incubated with macroGP, SA-macroGP, mesoGP, or SA-mesoGP (5, 2.5, or 0 mg) in 500 μL volumes at 37°C with gentle agitation for 1 h. Suspensions were centrifuged at 2,300 × *g* for 1 min to settle the GP particles. CFPs present within the supernatant (24 μL) were mixed with 5× loading buffer, separated via SDS-PAGE, and stained with Sypro Ruby (ThermoFisher). Three biological replicates of MRSA CFP were collected and subjected to GP adsorption assays. Two technical replicates for each CFP-GP adsorption assay were analyzed via SDS-PAGE and quantified using the ImageJ densitometry software program (NIH).

### α-Hemolysin (HLA) Toxin Adsorption and RRBC Hemolysis Assays

HLA is a 33.2 kDa protein that oligomerizes into a heptameric pore-forming toxin with a 100 Å solvent-filled channel (Bhakdi and Tranum-Jensen, 1991; Bhakdi et al., 1996; Song et al., 1996; Gouaux et al., 1997; Gouaux, 1998). With minor modifications to the methods described by Ragle et al. (Ragle and Wardenburg, 2009), HLA (100 nM) (H9395; Sigma-Aldrich, St. Louis, MO, United States) was incubated with 10, 5, 1, 0.75, 0.5, and 0.25 mg/mL macroGP, SA-macroGP, mesoGP, or SA-mesoGP in 0.9% NaCl (w/v; saline) for 1 h at 37°C with agitation (170 rpm). After incubation, the GP was pelleted by centrifugation (13,200 × *g* for 1 min), and the supernatant from each mixture (50 μL) was mixed with rabbit red blood cells (RRBC; Cat. IRBRBC10ML, Innovative Research, Novi, MI, United States) (final concentration 12.5% v/v) and statically incubated at 20°C for 1 h. Two positive controls, 1% Triton X-100 and HLA only, for 100% RRBC lysis and a GP only negative control to ensure lack of GP-mediated RRBC lysis were included in all experiments. After brief centrifugation to pellet the intact RRBCs, the OD_475_ of the recovered supernatants was measured. The percentage of hemolytic activity was determined by comparing the supernatant absorbance for all conditions tested to an equivalent number of RRBC lysed with 100 nM HLA. All RRBC assays were performed and analyzed in triplicate.

### Streptolysin-O (SLO) Toxin Adsorption and HRBC Hemolysis Assays

SLO is a 67 kDa monomeric protein that forms a large ring-like toxin that consists of 25–80 monomers (Bhakdi et al., 1996; Feil et al., 2014). Similar to the methods described for HLA adsorption by GP, the ability of GP to adsorb SLO was tested by pre-incubating the toxin with GP, followed by addition to human red blood cells (HRBC). To overcome the instability of SLO in the presence of oxygen, SLO (400 U/mL) (S5265; Sigma-Aldrich, St. Louis, MO, United States) was pre-incubated with the reducing agent 1,4-dithiothreitol (DTT; 0.5 mM) in phosphate-buffered saline (PBS, pH 7.4) for 30 min at 37°C. The SLO-DTT mixtures (hereafter referred to as SLO) were mixed with 10 mg/mL of macroGP, SA-macroGP, mesoGP, or SA-mesoGP. The GP and SLO were statically incubated at room temperature for 20 min so that GP could naturally sediment, while SLO would remain in suspension. The supernatant from each mixture (500 μL) was mixed with HRBCs (final concentration 2% v/v) and statically incubated at 37°C for 30 min. One unit of SLO will cause lysis of a 2% HRBC suspension at 37°C for 30 min (S5265; Sigma-Aldrich, St. Louis, MO, United States). Two positive controls, 1% Triton X-100 and SLO only, for 100% HRBC lysis and a GP only negative control to ensure lack of GP-mediated HRBC lysis were included in all experiments. After brief centrifugation to pellet the intact HRBCs, the OD_475_ of the recovered supernatants was measured. The percentage of hemolytic activity was determined by comparing the supernatant absorbance for all conditions tested to an equivalent number of HRBC lysed with SLO. All HRBC assays were performed and analyzed in triplicate.

### Bacterial Cell Adsorption Assays

To assess bacterial cell adsorption properties of macroGP, SA-macroGP, mesoGP, and SA-mesoGP, MRSA cultures were added to suspensions of GP. Mid-logarithmic phase MRSA cultures were resuspended in 0.9% NaCl (w/v; saline) and adjusted to an OD_600_ of 0.1 (1–3 × 10^7^ CFU/mL). The MRSA suspension (1 mL) was added to 10, 5, and 0 mg of macroGP, SA-macroGP, mesoGP, or SA-mesoGP, and subsequently incubated at 37°C with gentle agitation for 1 h. Each sample was filtered using a 5-μm cellulose syringe filter (Sigma-Aldrich) to separate freely suspended MRSA from the GP. The first “flow-through” was collected, and an additional 1 mL saline was passed through the filter, freeing any MRSA cells that were stuck in the filter or loosely adhered to the GP. The first and second flow-through collections were subjected to serial dilutions and plated in duplicate on TSA. After the TSA plates were incubated at 37°C for 14–18 h, CFU/mL for each GP-MRSA co-incubation was determined.

### Antibiotic Adsorption Assays

Vancomycin (VAN; 40 μg/mL; Sigma-Aldrich) or anhydrotetracycline (ATC; 50 μg/mL; Sigma-Aldrich) was mixed with 1 mg/mL macroGP, SA-macroGP, mesoGP, or SA-mesoGP in saline for 1 h at 37°C with agitation (170 rpm). After incubation, the GP was pelleted by centrifugation, and then the supernatant from each mixture was removed and diluted 10-fold into cation-adjusted Mueller Hinton Broth (CAMHB; pH 7.4). The minimum inhibitory concentration of VAN and ATC against MRSA in saline was 4 and 5 μg/mL, respectively. Supernatants from GP-adsorbed antibiotics, control antibiotic solutions not subjected to GP adsorption, and control GP only supernatants were statically incubated with mid-logarithmic phase MRSA (10^5^ CFU/mL) at 37°C for 24 h. The OD_600_ was measured, and control antibiotic only values were normalized to 100% inhibition.

### Statistical and Quantitative Analyses

We used one-way non-parametric (Kruskal-Wallis) analysis of variance (ANOVA) analyses with Dunn’s *post hoc* tests to assess statistical significance. Because statistical analyses were employed to compare pore size (macroGP vs mesoGP) and surface chemistry (non-coated GP vs SA-coated GP) impacts on whole cell and CFP adsorption, significance comparing SA-macroGP and SA-mesoGP, SA-macroGP and macroGP, SA-mesoGP and mesoGP, and macroGP and mesoGP, were reported. All statistical analyses were performed using Prism eight GraphPad software (San Diego, CA, United States). A radar chart (Microsoft Excel) graphically represents the relative performance metrics of macroGP, SA-macroGP, mesoGP, and SA-mesoGP materials.

## Results

### Characterization of GPs

**FIGURE 1:**
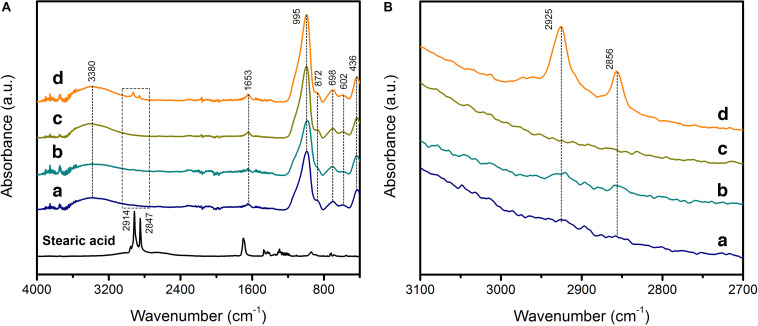
Fourier transform-infrared (FT-IR) spectra of stearic acid (SA) with GPs before and after surface modification for macroGP (a, dark blue), SA-macroGP (b, teal), mesoGP (c, olive), and SA-mesoGP (d, orange). **(A)** FT-IR 400–4000 cm^–1^ spectral region with 2700–3100 cm^–1^ spectral region marked with a dashed rectangle. **(B)** FT-IR 2700 – 3100 cm^–1^ spectral region.

The powder X-ray diffraction (PXRD) patterns of macroGP and mesoGP showed similar broad, featureless humps centered around 27–30° in 2θ (Supplementary Figure 1), indicating that both are non-crystalline geopolymeric materials (Davidovits, 1991). After modification with stearic acid (SA), the PXRD patterns of SA-macroGP and SA-mesoGP were similar to their parent materials, indicating that geopolymer structure was not affected by the surface modification experiments (Supplementary Figure 1). The products before and after modification were further characterized by FT-IR spectroscopic studies (Figure 1). MacroGP and mesoGP exhibited the characteristic GP spectra with bands at 436, 602, 698, 872, and 995 cm^–1^ in the low energy region (400–1000 cm^–1^) (Figure 1A), thereby confirming the PXRD analyses (Lecomte et al., 2003). The 436 cm^–1^ bands are due to the bending of T–O–T (*T* = Al or Si) links, while the 602 and 698 cm^–1^ bands are assigned to the symmetric stretching of T–O–T (Fontaine et al., 2003; Lecomte et al., 2003). The main broad band at 995 cm^–1^ revealed asymmetric stretching of T–O–T, while the 872 cm^–1^ band could be due to Si–O bond stretching or OH bending of Si–OH (Lee and van Deventer, 2003). In addition, the bands at 1653 and 3380 cm^–1^ originated from water molecules (Yuan et al., 2016). After modification, the FT-IR spectra of the SA-macroGP and SA-mesoGP products maintain the same features of the parent materials (Figure 1A) and yet show two weak additional bands at 2856 and 2925 cm^–1^ (Figure 1B). Those bands are associated with C–H asymmetric and symmetric stretching modes, respectively, of methyl or methylene groups in SA (Coates, 2000), indicating the presence of SA in the two products. The peaks were more prominent for SA-mesoGP than SA-macroGP (Figure 1B), which suggests a larger amount of SA in the former.

**FIGURE 2:**
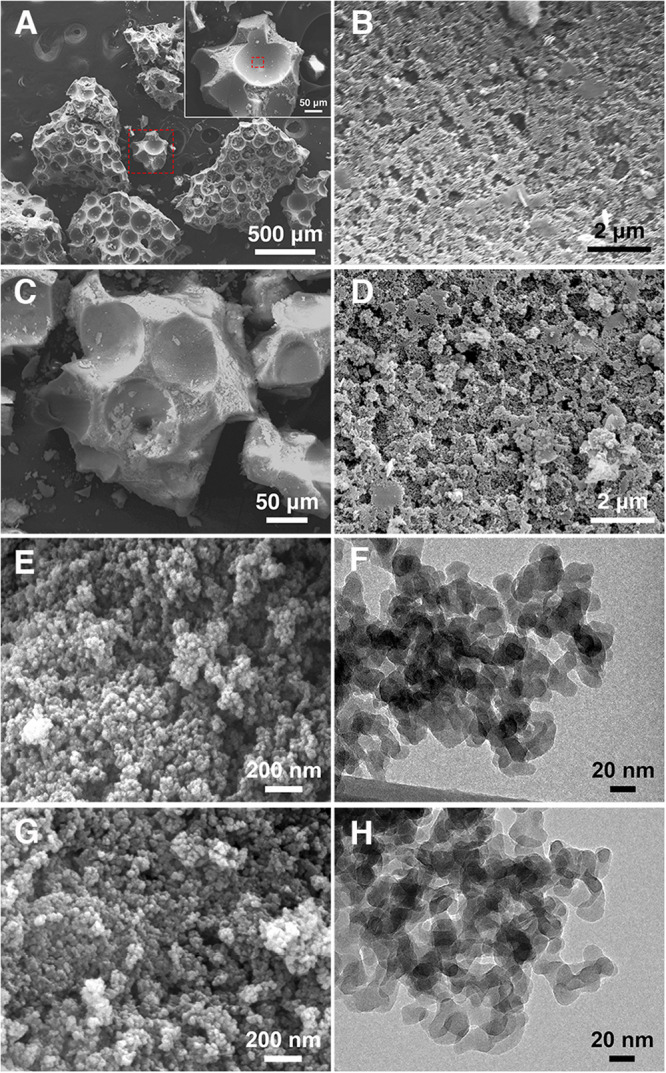
SEM and TEM images of the GPs. **(A)** MacroGP SEM image at low magnification (500 μm scale bar) with hatched red square representing the higher magnification inset (50 μm scale bar). **(B)** MacroGP SEM image at high magnification (2 μm scale bar) representing the inset area marked with a hatched red square in the panel A inset. **(C)** SA-macroGP SEM image (50 μm scale bar). **(D)** SA-macroGP SEM image at high magnification (2 μm scale bar) representing the inset area marked with hatched red square in the panel C inset. **(E)** SEM image of mesoGP (200 nm scale bar). **(F)** TEM image of mesoGP (20 nm scale bar). **(G)** SEM image of SA-mesoGP (200 nm scale bar). **(H)** TEM image of SA-mesoGP (20 nm scale bar).

Scanning electron microscopy (SEM) imaging revealed macroGP with seemingly discrete spherical pores with pore diameters in the range of in the 50–200 μm (Figure 2A). A close-up image of the pore wall (Figure 2B; a magnified micrograph of the area boxed in red in the *A* inset) revealed additional, smaller pores ranging from 200–500 nm (Table 1). This pore size range is in agreement with our previous results with paraffin oil (Seo et al., 2016). The smaller pores are due to the nanoscopic biphase formation between the inorganic geopolymer component and the organic paraffin oil component. The much larger spherical pores coexist because after the biphase formation, any excess amount of paraffin oil remains in the mixture as large oil droplets (Moshoeshoe et al., 2017). After curing the geopolymer component, oil extraction leaves the two types of pore structures in the product. In Figures 2C,D, the SA-macroGP exhibited the same morphology as its parent macroGP, as we expect that the surface modification would not change the morphology of the inorganic geopolymer.

**TABLE 1:** CHN analyses and pore properties of GP products.

**Sample**	**Carbon (wt%)^a^**	**Hydrogen (wt%)^a^**	**Nitrogen (wt%)^a^**	**SSA_*BET*_ (m^2^/g)**	**Number^b^ of SA/nm^2^**	**BJH pore width (nm)**	**Macropore size range^c^**
mesoGP	0.06 (±7)	1.27 (±19)	−0.04 (±3)	137	0.0	31	–
SA-mesoGP	3.54 (±1)	1.42 (±6)	−0.01 (±3)	–	0.7	–	–
macroGP	0.40 (±3)	0.97 (±23)	−0.09 (±8)	36	0.0	26	200–500 nm; 50–200 μm
SA-macroGP	0.66 (±3)	0.44 (±9)	−0.05 (±6)	–	0.5	–	–

*^*a*^Standard deviations in the elemental analysis results are shown in parentheses. ^*b*^Based on the carbon content and total surface area (SSA_*BET*_) of GP. ^*c*^Observed via SEM.*

SEM imaging revealed mesoGP with a uniform morphology of aggregates (Figures 2E,G). Transmission electron microscopy (TEM) imaging indicated that the aggregates are composed of strongly interconnected primary nanoparticles with 20–30 nm in diameter (Figures 2F,H). Textural porosity is apparent between the nanoparticles. No appreciable change of morphology and nanostructure was observed after the surface modification by SA (Figures 2G,H).

Elemental CHN analysis showed that mesoGP contained only a limited amount of carbon (0.06 wt%) (Table 1), which is anticipated because no organic compounds were used during the synthesis process. However, after modification, 3.54 wt% of carbon was present within the SA-mesoGP (Table 1), which is consistent with FT-IR analysis indicating that the surface of mesoGP was successfully modified by the SA (Figure 1). The total surface area for mesoGP is as high as 137 m^2^/g, and thus, the surface coverage by SA is about 0.7 molecule/nm^2^ (Table 1). Despite the extraction treatment with hot hexane, macroGP showed slightly higher carbon content (0.40 wt%) than mesoGP (Table 1). After modification, the carbon content of SA-macroGP increased to 0.66 wt%, which is equivalent to the surface coverage by SA of about 0.5 molecule/nm^2^ with the total surface area of 36 m^2^/g (Table 1). The GP samples lacked nitrogen and had only small amounts of hydrogen (Table 1). Notably, BJH analyses revealed that the average pore size of mesoGP was 31 nm (Table 1), corroborating the presence of textural porosity observed in the TEM studies (Figure 2). While macroGP exhibited large pore sizes in the range of 50–200 μm and 200–500 nm (Figure 2), they also contained a small number of mesopores with an average pore size of 26 nm (Table 1).

### Geopolymer Adsorption of MRSA Culture Filtrate Proteins

**FIGURE 3:**
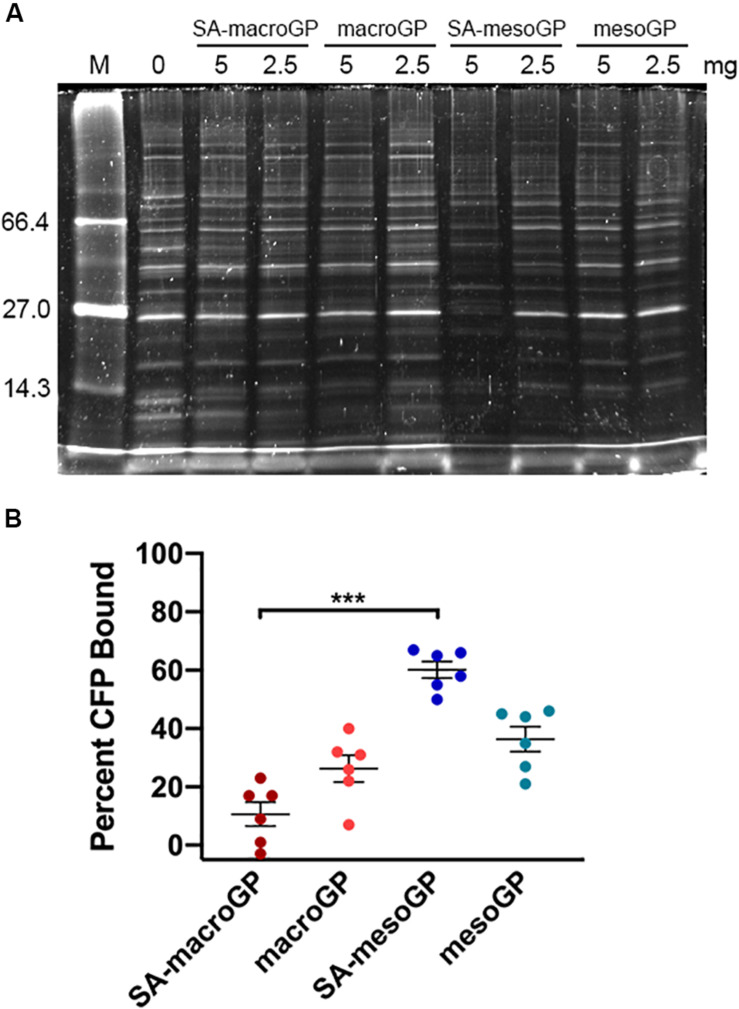
**(A)** Representative SDS-PAGE gel of MRSA CFP (50 μg/mL) with and without preincubation with 10 mg/mL GP. MW standards are shown in lane *M* and are indicated in kDa. **(B)** Densitometry-based quantitation of total CFPs adsorbed by GPs. Individual symbols represent the values for each replicate. Each line represents the mean of three biological replicates, each with two technical replicates ± SEM of CFP adsorption for each GP, compared to the control (no GP preincubation; 0% adsorption). ***P < 0.001; Kruskal-Wallis ANOVA with Dunn’s *post hoc* test comparing all four GP samples to each other.

MRSA secretes a multitude of virulence factors to aid in pathogenesis and evasion of the host immune system. Thus, removal of secreted MRSA proteins (represented by CFPs) from the SSTI site could reduce pathogenesis, since inhibiting virulence factors attenuates MRSA infection (Patel et al., 1987; Tang et al., 2014). GP and MRSA CFPs were co-incubated, and unbound CFPs were separated and analyzed via SDS-PAGE (Figure 3A). Visualization of the CFP banding patterns revealed that SA-mesoGP adsorbed the most CFPs (Figure 3A), which was confirmed by densitometry quantitation (Figure 3B). In general, all GPs adsorbed smaller MW CFPs (<15 kDa), while SA-mesoGP and mesoGP also adsorbed some large CFPs (>100 kDa) (Figure 3 and Supplementary Figure 2). In comparison, all four GPs adsorbed similar amounts of total human serum proteins, as confirmed by densitometry quantitation (Supplementary Figure 3).

### Geopolymer Adsorption of HLA

**FIGURE 4:**
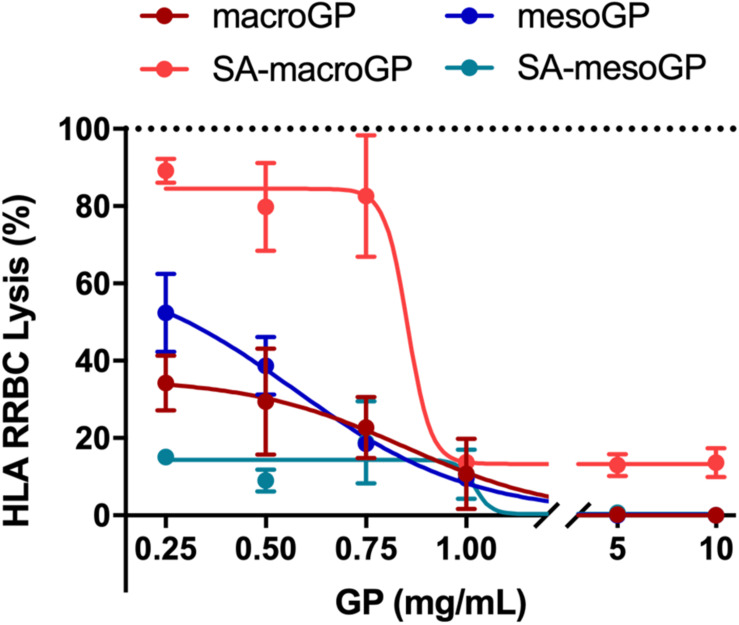
Percentage of RRBC lysis after HLA (100 nM) incubation with increasing GP concentrations. Dotted line represents the lysis percentage (100%) of the HLA only control. Each point represents the mean ± SEM of triplicate experiments for each GP, normalized to the HLA only control (100% lysis), with best-fit non-linear regression lines shown.

*S. aureus* secretes HLA monomers which bind to and oligomerize within eukaryotic cell membranes causing lysis (Bhakdi and Tranum-Jensen, 1991; Song et al., 1996). When produced at sufficient concentrations, the pore-forming HLA toxin serves as a key virulence factor and can permeate any eukaryotic cell, causing cytotoxicity or cytolysis. To determine if GP could adsorb and remove HLA from solution, GP and toxin co-incubations were performed prior to RRBC exposure. At 1 mg/mL, all GP protected RRBC from HLA-mediated lysis (Figure 4). At higher concentrations (5 and 10 mg/mL), macroGP, mesoGP, and SA-mesoGP completely protected RRBC from HLA, while increased concentrations of SA-macroGP did not further alter RRBC lysis (Figure 4). SA-mesoGP adsorbed significantly more HLA than mesoGP at 0.5 and 0.25 mg/mL, and SA-macroGP adsorbed the least HLA of all GPs (Figure 4). Increasing concentrations of mesoGP and macroGP steadily increased HLA adsorption and decreased RBBC lysis (Figure 4). The results indicate that the adsorption capacity of the GP for HLA monomers was at least 0.66 mg/g. The minimum adsorption capacity was estimated by dividing the amount of the HLA (3.3 μg/mL, or equivalently 100 nM) by the minimum weight of the GP required to observe complete HLA adsorption (5 mg/mL). It is possible that low levels of SA modification (Figure 1 and Table 1) could contribute to reduced performance of SA-macroGP.

### Geopolymer Adsorption of SLO

**FIGURE 5:**
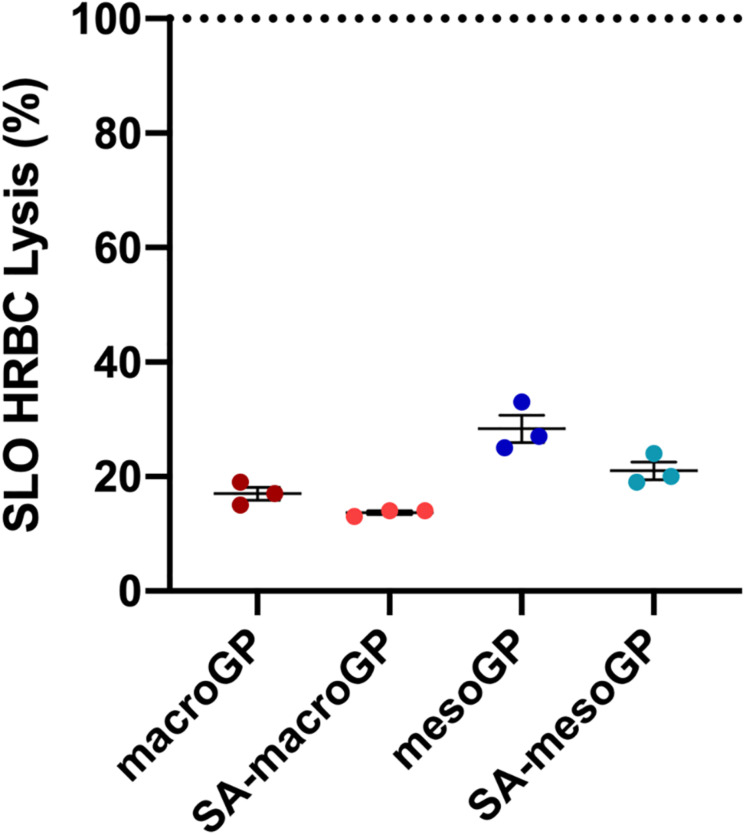
Percentage of HRBC lysis after SLO (400 U/mL) incubation with GPs (10 mg/mL). Individual symbols represent the value for each biological replicate. Each line represents the mean of three biological replicates, each with two technical replicates ± SEM of HRBC lysis for each GP, normalized to the SLO only control. Dotted line represents the lysis percentage of the SLO only control. All four GP samples adsorbed SLO similarly; Kruskal-Wallis ANOVA with Dunn’s *post hoc* test.

SLO, a potent cytolytic toxin produced and secreted by group A streptococci, binds to eukaryotic cell membranes, oligomerizes to generate large pores, and causes cell lysis (Palmer et al., 1998). Sublytic concentrations of SLO produce a multitude of subtle effects on targeted cells, including macrophage apoptosis leading to enhanced bacterial survival and virulence and diminished host cytokine responses (Fontaine et al., 2003; Timmer et al., 2009). To determine if GP could bind and neutralize SLO, GP and SLO co-incubations were performed prior to HRBC exposure and cytotoxicity determinations. At 10 mg/mL, all GP adsorbed SLO and protected HRBC from SLO-mediated cytolysis (Figure 5). When comparing GP, macroGP was the superior pore size for adsorption of SLO and SA modification aided SLO binding with HRBC protection (Figure 5).

### Geopolymer Binding of MRSA Cells

**FIGURE 6:**
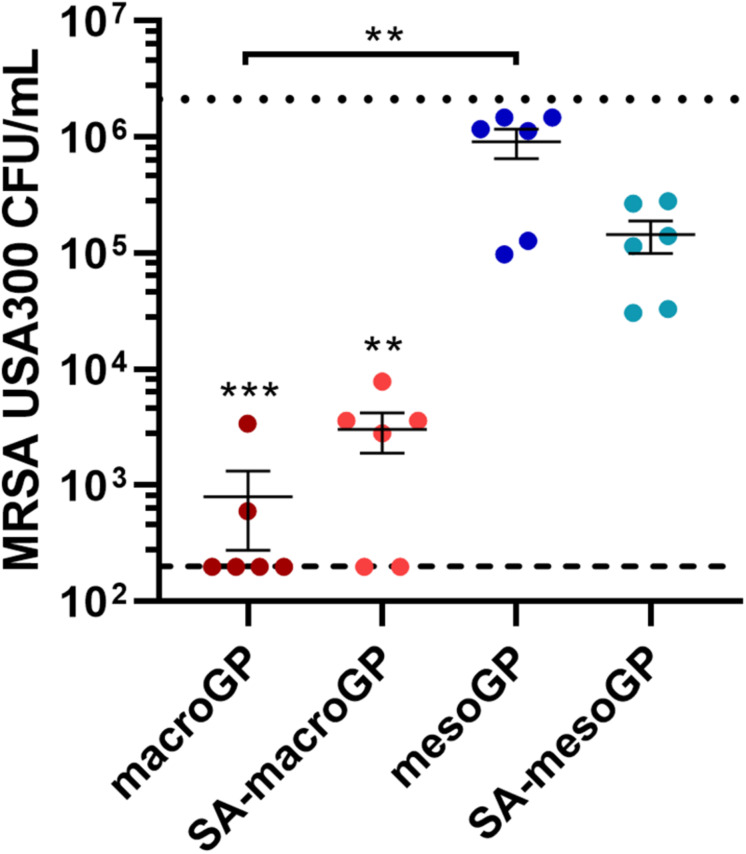
MRSA USA300 quantitation after incubation with 10 mg/mL of each GP for 1 h in saline. Individual symbols represent the value for each replicate. Each line represents the mean of three biological replicates, each with two technical replicates ± SEM of triplicate experiments. Dotted line represents the saline only control. Dashed line represents the limit of detection. **P < 0.01; ***P < 0.001; Kruskal-Wallis ANOVA with Dunn’s *post hoc* test comparing all four GP samples to each other and to the saline-only control.

To determine if GP was capable of physically removing bacterial cells from suspension, GP was incubated with mid-logarithmic phase MRSA for 1 h and non-adherent cells were quantified. Although macroGP and SA-macroGP (10 mg/mL) significantly adsorbed MRSA (*P* < 0.001), macroGP adsorbed the most MRSA cells, reducing average cell counts by 3.4 log_10_ compared to incubation with saline only (Figure 6). These results indicate that MRSA cell binding is greatest with macroGP, followed by SA-macroGP and SA-mesoGP. MesoGP poorly adsorbed MRSA cells (Figure 6).

### Geopolymer Adsorption of Antibiotics

**FIGURE 7:**
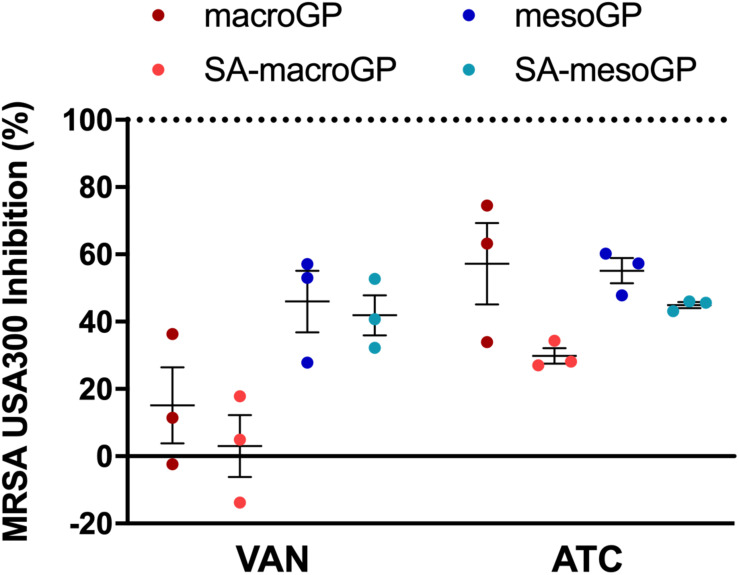
Percentage of MRSA inhibition after antibiotic pre-incubation with GP. Antibiotics, VAN (4 μg/mL) and ATC (5 μg/mL), were preincubated with GP (1 mg/mL) in saline for 1 h at 37°C. Dotted line represents the percent inhibition (100%) of antibiotics only. Individual symbols represent the value for each biological replicate. Each line represents the mean of three biological replicates, each with two technical replicates ± SEM normalized to the antibiotic only control. All four GP samples adsorbed antibiotics similarly; Kruskal-Wallis ANOVA with Dunn’s *post hoc* test.

In practical applications involving GP, adsorption could impact clinical applications and treatment timing. Water-soluble (vancomycin; VAN) and water-insoluble (anhydrotetracycline; ATC; 5 μg/mL) antibiotics were pre-incubated with GP for 1 h, and the supernatant was incubated with MRSA USA300 in a broth microdilution assay. As indicated by reduced inhibitory activity, VAN (MIC; 4 μg/mL) was adsorbed by all GP types (Figure 7). VAN was adsorbed less by mesoGP, with non-adsorbed VAN causing 42–46% MRSA inhibition, compared to macroGP which greatly limited VAN-mediated MRSA inhibition to 3–15% (Figure 7). The amount of water-insoluble ATC remaining after macroGP and mesoGP adsorption resulted in ∼56% MRSA inhibition, while remaining ATC, after SA-macroGP and SA-mesoGP adsorption, resulted in ∼30 and 45% MRSA inhibition, respectively (Figure 7).

## Discussion

In the age of antibiotic resistance, there is a tremendous need for novel ways to prevent and treat infections and minimize further spread and evolution of multidrug resistant bacteria. In the past few decades, aluminosilicate-based removal of harmful toxins from the environment has been studied, and the results are promising. GPs could serve as controllable, cost-effective, and synthetic aluminosilicate materials for minimizing and removing pathogenic material, such as toxins, from patient infection sites, improving patient outcomes and reducing reliance on antibiotics. While the adsorption of antibiotics with GPs is a potential concern for infection treatment, GP-mediated antibiotic removal in hemodialysis cases (Holubek et al., 2008) and in poultry, agricultural, environmental, and aquaculture settings, where pharmaceutical compounds are considered emerging pollutants (McClellan and Halden, 2010; Done and Halden, 2015; Priya and Radha, 2017; Tang et al., 2017), and in drug delivery applications (Lopes et al., 2014) could be greatly beneficial.

To assess feasibility, we synthesized four GP materials and characterized adsorptive properties with clinically and pathologically relevant biomolecules and bacterial cells. Using quantitative techniques, the adsorption efficacies of different GPs were tested against different biological adsorbates. These GPs have anionic aluminosilicate surfaces due to their zeolite-like chemical composition and are expected to adsorb biomolecules due to their innate, negatively charged surfaces which attract polar functional groups of biomolecules and bacterial cells (Fosso-Kankeu and Mishra, 2017). With the surface modification by SA, we anticipated that SA-macroGP and SA-mesoGP would have enhanced binding of hydrophobic biomolecules or proteins with partially exposed side chains of hydrophobic amino acids due to the increased hydrophobicity of the GP surface. Stearic acid was selected for its low cytotoxicity, thereby enabling studies focused on the pore and surface characteristics of the materials in absence of any innate antibacterial properties of the surface-modifying organic molecules.

MacroGP and mesoGP were prepared independently in order to vary the pore characteristics. MacroGP was synthesized using paraffin oil as the emulsion template, generating mainly macropores with pore diameters ranging between 50 and 200 μm. Such macroGP pore architecture was suitable for adsorbing large biomaterials or small microorganisms such as MRSA bacterial cells (∼1 μm) and would help diffusion of adsorbates into the pores, but the relatively low surface area of only 36 m^2^/g (Table 1) could limit adsorption capacity. In contrast, mesoGP had a higher surface area (137 m^2^/g) (Table 1), theoretically resulting in a higher adsorption capacity. However, with pore throats between 10–80 nm, mesoGP would not be expected to adsorb the relatively large bacterial cells or protein complexes, but rather, would adsorb small proteins and molecules.

**FIGURE 8:**
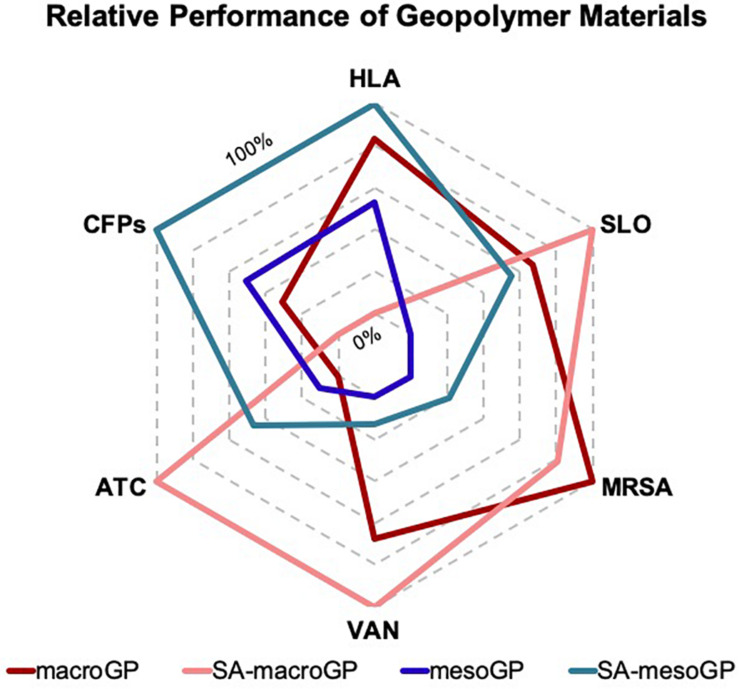
Graphical representation of the relative performance metrics of macroGP, SA-macroGP, mesoGP, and SA-mesoGP materials. For CFPs, HLA, SLO, and MRSA, relative performance was determined by GP-mediated protein, toxin, or bacterial adsorption efficiency. For ATC and VAN, relative performance was determined by retention of antibiotic activity after exposure to GP. Relative performance metrics were based on the following GP concentrations and respective biological adsorbates: 0.25 mg/mL GP – HLA; 10 mg/mL GP – SLO and MRSA; 1 mg/mL GP – VAN and ATC. The most desirable GP features are on the outermost axis (100%) of the radar graph, while the least desirable GP performances are on the innermost axis (0%). Each pentagon axis beyond the innermost pentagon ring represents enhanced relative performance metrics (20% per line).

Previous work has shown that interactions between the GP surface and the different adsorbates are highly dependent on adsorbate structure, properties, and molecular interactions (Huwig et al., 2001; Yu et al., 2013; Fosso-Kankeu and Mishra, 2017). Figure 8 depicts the relative GP performance related to protein, toxin, or bacterial adsorption efficiency or retention of antibiotic activity after GP exposure. In general, SA-macroGP exhibited the most desirable relative performances associated with adsorption of MRSA cells and the SLO toxin and lack of adsorption of antibiotics (Figure 8). Although there is strong similarity between the shapes of the mesoGP and SA-mesoGP grid lines, SA-mesoGP displayed a greater relative performance in adsorbing MRSA CFPs and HLA toxin than meso-GP (Figure 8). These comprehensive results further support the idea that for each adsorbate, optimization of pore size and surface coating is the best method for creating an ideal adsorbent.

In summary, we determined that GPs adsorbed and physically removed several adsorbates, including large pore-forming toxins, MRSA proteins, and viable MRSA cells, from solution with different efficacies. Hence, these laboratory-synthesized GP materials could serve as cost-effective aluminosilicates for biomedical applications, physically removing harmful toxins and cells from infectious wound sites. Future optimization of GP with different modifications and pore sizes could influence adsorption and adsorption rates. Therefore, ongoing investigations of GP, modified GP, and GP-based products as non-selective or selective adsorbents (Sturino et al., 2015) and for incorporation into fibrous materials are warranted.

## Data Availability Statement

All datasets generated for this study are included in the article/Supplementary Material.

## Author Contributions

D-KS and SH conceived and supervised the research. JP, SC, D-KS, and SH designed the experiments. JP, SC, NI, and CG performed the experiments. JP, SC, NI, CG, D-KS, and SH analyzed the experimental results. All authors participated in writing and editing the manuscript.

## Conflict of Interest

The authors declare that the research was conducted in the absence of any commercial or financial relationships that could be construed as a potential conflict of interest.
