# Supplementary material for: Synthesized Geopolymers Adsorb Bacterial Proteins, Toxins, and Cells
Source: Front Bioeng Biotechnol. 2020 Jun 3;8:527. doi: 10.3389/fbioe.2020.00527 (PMC7283576; doi:10.3389/fbioe.2020.00527)
Supplement: Supplementary file 1 [file Data_Sheet_1.docx]

Synthesized geopolymers­ adsorb bacterial proteins, toxins, and cells

**John Popovich, BS^1†^, Shaojiang Chen, BS^2^, Natalie Iannuzo, BS^2,3^, Collin Ganser^3,4^, Dong-Kyun Seo, PhD^2,5^*, and Shelley E. Haydel, PhD^1,3,6^***

^1^Center for Immunotherapy, Vaccines and Virotherapy, The Biodesign Institute, Arizona State University, Tempe, AZ, USA

^2^School of Molecular Sciences, Arizona State University, Tempe, AZ, USA

^3^School of Life Sciences, Arizona State University, Tempe, AZ, USA

^4^School of Earth and Space Exploration, Arizona State University, Tempe, AZ, USA

^5^Center for Molecular Design and Biomimetics, The Biodesign Institute, Arizona State University, Tempe, AZ, USA

^6^Center for Bioelectronics and Biosensors, The Biodesign Institute, Arizona State University, Tempe, AZ, USA

*** Correspondence:**

Shelley E. Haydel
[Shelley.Haydel@asu.edu](mailto:Shelley.Haydel@asu.edu)

Don Seo

[DSeo@asu.edu](mailto:DSeo@asu.edu)

^†^Current address: Midwestern University, Chicago College of Osteopathic Medicine, Downers Grove, IL, USA

Running Title: Geopolymers as bacterial biomolecule adsorbents

Keywords: aluminosilicate, geopolymer, nanoporous, adsorption, adsorbent, toxin removal, bacteria

**Supplementary Materials**

**Materials and Methods (Supplementary Materials)**

**Geopolymer adsorption of serum proteins**

Human serum (diluted 0.5% in sterile dH_2_O) (500 μg/mL) was incubated with macroGP, SA-macroGP, mesoGP, or SA-mesoGP (5 or 0 mg) in 500 μL volumes at 37°C with gentle agitation for 1 h. Suspensions were centrifuged at 2,300 x *g* for 1 min to settle the GP particles. Serum proteins remaining within the supernatant (24 μL) were mixed with 5X SDS-PAGE loading buffer (6 μL), separated via SDS-PAGE, stained with Sypro Ruby (ThermoFisher), and analyzed with ImageJ densitometry (NIH).

**Results (Supplementary Materials)**

**Geopolymer adsorption of serum proteins**

Many proteins exist in human serum, thereby potentially competing for binding if GPs are incorporated into topical treatments for SSTIs. To assess serum protein adsorption, human serum (0.5%; ~500 μg/mL) was co-incubated with GPs (10 mg/mL) and unbound serum proteins were analyzed with SDS-PAGE and densitometry, revealing that the GPs adsorbed 10-20% of the serum proteins present (Figure S3).

**Supplementary Table 1.** Chemically-defined medium (CDM) for MRSA growth.

| Ingredient | Amount (per Liter) |
| --- | --- |
| Amino acids |  |
| l-glutamic acid | 100 mg |
| l-serine | 30 ng |
| l-methionine | 3 mg |
| l-tyrosine | 50 mg |
| l-alanine | 60 mg |
| l-lysine | 50 mg |
| l-threonine | 30 mg |
| l-phenylalanine | 40 mg |
| l-histidine | 20 mg |
| glycine | 50 mg |
| l-tryptophan | 10 mg |
| l-isoleucine | 30 mg |
| l-valine | 80 mg |
| l-leucine | 90 mg |
| l-aspartic acid | 90 mg |
| l-arginine | 50 mg |
| l-proline | 80 mg |
| l-cysteine | 20 mg |
| l-asparagine | 30 mg |
| l-glutamine | 30 mg |
| Sugar |  |
| Dextrose anhydrous | 5 g |
| Salts |  |
| K_2_HPO_4_ | 7 g |
| KH_2_PO_4_ | 2 g |
| Na citrate 2H_2_O | 0.4 g |
| MgSO_4_ | 0.05 g |
| (NH_4_)_2_SO_4_ | 1 g |
| Vitamins |  |
| Thiamine HCl | 5 mg |
| Nicotinic acid | 6 mg |
| Biotin | 5 ng |
| Ca pantothenate | 0.25 mg |
| Nitrogenous bases |  |
| Guanine | 5 mg |
| Cytosine | 5 mg |
| Uracil | 5 mg |
| Thymine | 10 mg |

**
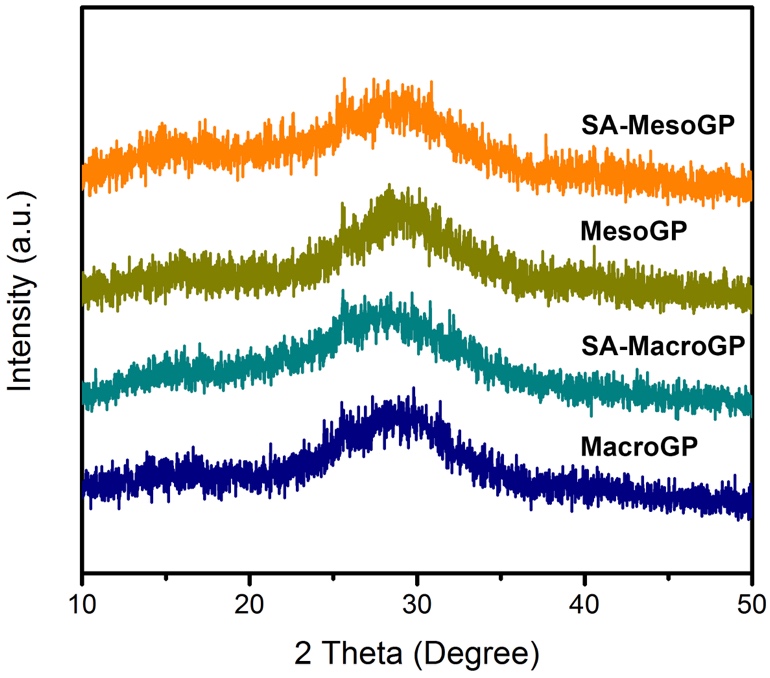
**

**Supplementary Figure 1.** Powder X-ray diffraction patterns of macroGP (dark blue), SA-macroGP (teal), mesoGP (olive), and SA-mesoGP (orange).

**
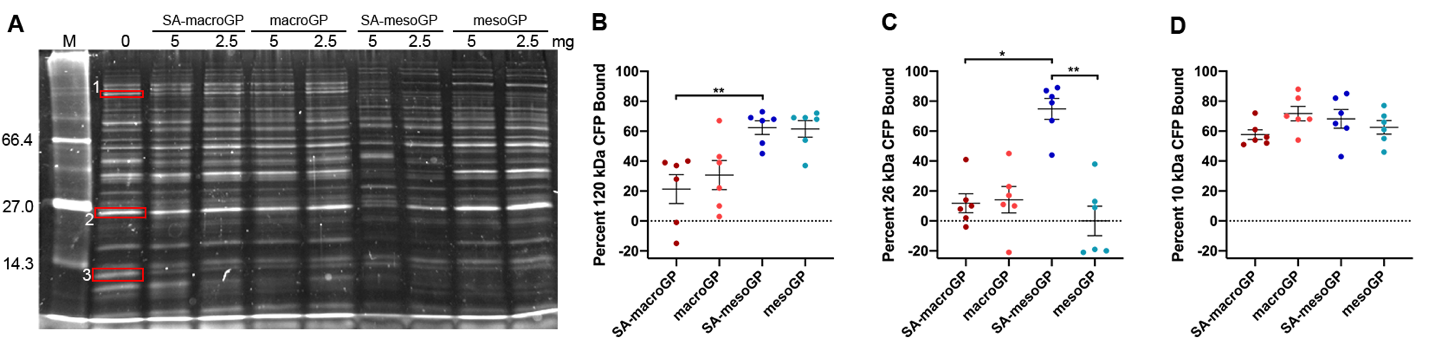
**

**Supplementary Figure 2.** (**A**) Representative SDS-PAGE gel of MRSA CFP (50 μg/mL) with and without preincubation with GP. MW standards are shown in lane *M* and are indicated in kDa. The three specific CFPs analyzed are shown with red rectangles in lane 2. Specific large (~120 kDa), medium-sized (~26 kDa), and small (~10 kDa) CFP proteins are labeled 1, 2, and 3, respectively. Densitometry-based quantitation of (**B**) ~120 kDa, (**C**) ~26 kDa, and (**D**) ~10 kDa CFPs adsorbed by 5 mg of the respective GPs. Each line represents the mean of three biological replicates, each with two technical replicates, ± SEM of CFP adsorption for each GP, compared to the control (no GP preincubation; 0% adsorption). *, *P* < 0.05; **, *P* < 0.01; Kruskal-Wallis ANOVA with Dunn’s post-hoc test comparing all four GPs to each other.


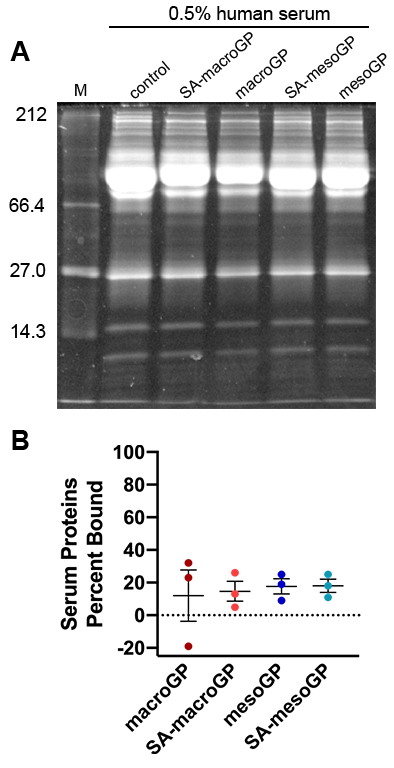


**Supplementary Figure 3.** (**A**) Representative SDS-PAGE gel of 0.5% human serum (500 μg/mL) with and without preincubation with 10 mg/mL GP. MW standards are shown in lane *M* and are indicated in kDa. The control lane represents serum incubated without GPs. (**B**) Densitometry-based quantitation of total serum proteins adsorbed by GPs. Individual symbols represent the values for each biological replicate. Each line represents the mean of three biological replicates ± SEM of serum protein adsorption for each GP, compared to the control (no GP preincubation; 0% adsorption).
